# Multi-spatiotemporal analysis of changes in mangrove forests in Palawan, Philippines: predicting future trends using a support vector machine algorithm and the Markov chain model

**DOI:** 10.14324/111.444/ucloe.000057

**Published:** 2023-04-28

**Authors:** Cristobal B. Cayetano, Lota A. Creencia, Emma Sullivan, Daniel Clewely, Peter I. Miller

**Affiliations:** 1 College of Fisheries and Aquatic Sciences, Western Philippines University, Sta. Monica, Puerto Princesa City, Palawan, Philippines; 2 Remote Sensing Group, Plymouth Marine Laboratory, Prospect Place, Plymouth PL4 7QP, UK

**Keywords:** change detection, image classification, Landsat, land use/land cover, Markov chain model, spatial dynamics, support vector machine

## Abstract

Multi-temporal remote sensing imagery can be used to explore how mangrove assemblages are changing over time and facilitate critical interventions for ecological sustainability and effective management. This study aims to explore the spatial dynamics of mangrove extents in Palawan, Philippines, specifically in Puerto Princesa City, Taytay and Aborlan, and facilitate future predictions for Palawan using the Markov Chain model. The multi-date Landsat imageries during the period 1988–2020 were used for this research. The support vector machine algorithm was sufficiently effective for mangrove feature extraction to generate satisfactory accuracy results (>70% kappa coefficient values; 91% average overall accuracies). In Palawan, a 5.2% (2693 ha) decrease was recorded during 1988–1998 and an 8.6% increase in 2013–2020 to 4371 ha. In Puerto Princesa City, a 95.9% (2758 ha) increase was observed during 1988–1998 and 2.0% (136 ha) decrease during 2013–2020. The mangroves in Taytay and Aborlan both gained an additional 2138 ha (55.3%) and 228 ha (16.8%) during 1988–1998 but also decreased from 2013 to 2020 by 3.4% (247 ha) and 0.2% (3 ha), respectively. However, projected results suggest that the mangrove areas in Palawan will likely increase in 2030 (to 64,946 ha) and 2050 (to 66,972 ha). This study demonstrated the capability of the Markov chain model in the context of ecological sustainability involving policy intervention. However, as this research did not capture the environmental factors that may have influenced the changes in mangrove patterns, it is suggested adding cellular automata in future Markovian mangrove modelling.

## Introduction

Mangroves are a group of complex trees and shrubs that naturally inhabit the intertidal zones of coastal tropical and subtropical regions [1,2]. Although they can tolerate a wide range of salinity, from hypersaline waters exceeding 100 parts per thousand to lower salinities of 2 parts per thousand [3], they cannot compete reproductively with other terrestrial plants because the latter have a better adaptation to a higher-elevation environment [4]. Mangrove forests are one of the most important coastal ecosystems because they provide bio-productivity, for example, timber and firewood, and they provide protection from natural hazards and regulation of natural phenomena, for example, floods, storm erosion and salt intrusion [1,5,6]. They serve as a nursery and a habitat for biodiversity, for example, breeding and spawning [7–9], they are of socio-economic and cultural importance, for example, providing livelihoods, ecotourism, recreation and are of aesthetic importance [10,11], and help mitigate climate change, for example, carbon sequestration [10,12].

There are about 65 mangrove species around the world [13], of which at least 50% currently grow in the Philippines [14]. According to the Food and Agricultural Organization [15], Asia has more extensive mangrove forests than any other continent. The Philippines is considered one of the top biodiversity ‘hot spot’ countries in the world [16]. The Palawan Council for Sustainable Development Staff (PCSDS) [17] initially reported 27 mangrove species in Palawan. About 22.23% (56,261.3 ha) of the remaining mangrove forests in the Philippines are found in Palawan [18]. However, the ability of this ecosystem to colonise and maintain its spatial setting is increasingly being affected by anthropogenic disturbances [19]. Consequently, mangrove forest cover in the Philippines has decreased from approximately 500,000 ha in 1918 to about 120,000 ha by the end of 1995 [20,21]. Dodd and Ong reported that the two main contributing factors for this decline are overexploitation of raw product and coastal land use conversions (e.g., agriculture, residential settlements, industrial and aquaculture) [21]. Although recent estimates from the Department of Environment and Natural Resources (DENR) [22] suggest an increase in mangrove extent in 2003 (to 247,362 ha), this estimate is still much lower than the estimated area covered in the previous century.

Mangrove ecosystems form a complex structure (e.g., less accessible *Rhizophora*’s complex bifurcated and looping root structures), and the technical skills required and cost associated with taking forest samples make extensive in-situ sampling difficult. Thus, remote sensing techniques provide a convenient tool to map, assess and monitor the mangroves over large areas and can be used to detect change over time [23–25]. In the Philippines, the utilisation of remotely-sensed satellite data (e.g., [18]) has been incorporated into policy formulation and enforcement. However, mangrove-related projects in the country remain relatively scarce with only a few national and local mapping efforts focused on the classification and detection of changes in the mangrove’s extent, notably from the nominal years of 1990–2010 [26] and 2003–2013 [27]. Despite the low utilisation of mangrove remote sensing in the Philippines and the absence of projected data about how the remaining mangroves in the country will respond to the impacts of climate change, scientific interest in mitigating and controlling the magnitude of climate change’s impacts on mangrove ecosystems has increased in Southeast Asian countries [28]. The mangroves of Palawan have been protected under direct human inventions through the International Union for Conservation of Nature (IUCN) protected area Category I–IV [18] and the 1992 Republic Act No. 7611, commonly known as the Strategic Environmental Plan for Palawan Act (SEP Law) [29]; yet this unique ecosystem remains under threat due to climate change and the associated rising sea levels [18,30].

Several land use/land cover (LULC) techniques have been developed and utilised in the last three decades, which primarily aim to investigate the spatiotemporal changes of LULC patterns using satellite data to assist in ecological management and decision-making [31]. The parametric (e.g., maximum likelihood classifier [32]) and nonparametric (e.g., artificial neural networks [33]) classification algorithms can handle complex classification tasks [34]. To perform the classification using a supervised classification technique, training samples must be extracted, which can be time-consuming when using multi-temporal remotely sensed imagery. Unsupervised classification techniques have also been used to map mangrove extent and change over time, for example, using vegetation indices (e.g., the Normalised Difference Vegetation Index, the Mangrove Vegetation Index [35,36]) and clustering and threshold techniques (e.g., [37]). The Markov chain model [38,39] is one of many prediction techniques that are able to assess the LULC changes and make a projection of these changes in the future [40–43]. Understanding the patterns of change in mangrove geographic distribution and projecting the range of shifts in the future will link science to policy and decision-making processes for biodiversity conservation and management [44].

Through the Global Challenges Research Fund (GCRF) Blue Communities (BC), this research aims to: (1) develop a mapping approach to investigate the changes in mangrove extents in Palawan using multi-temporal Landsat imagery during the years 1988, 1993, 1998, 2003, 2008, 2013, 2018 and 2020; (2) determine the areal extent of change in mangrove forests in Palawan including the three case study areas of GCRF BC from 1988 to 2020; and (3) implement change projections of the mangrove forests in Palawan for 2030 and 2050 using a Markov chain model.

## Materials and methods

### Study area

**Figure 1:**
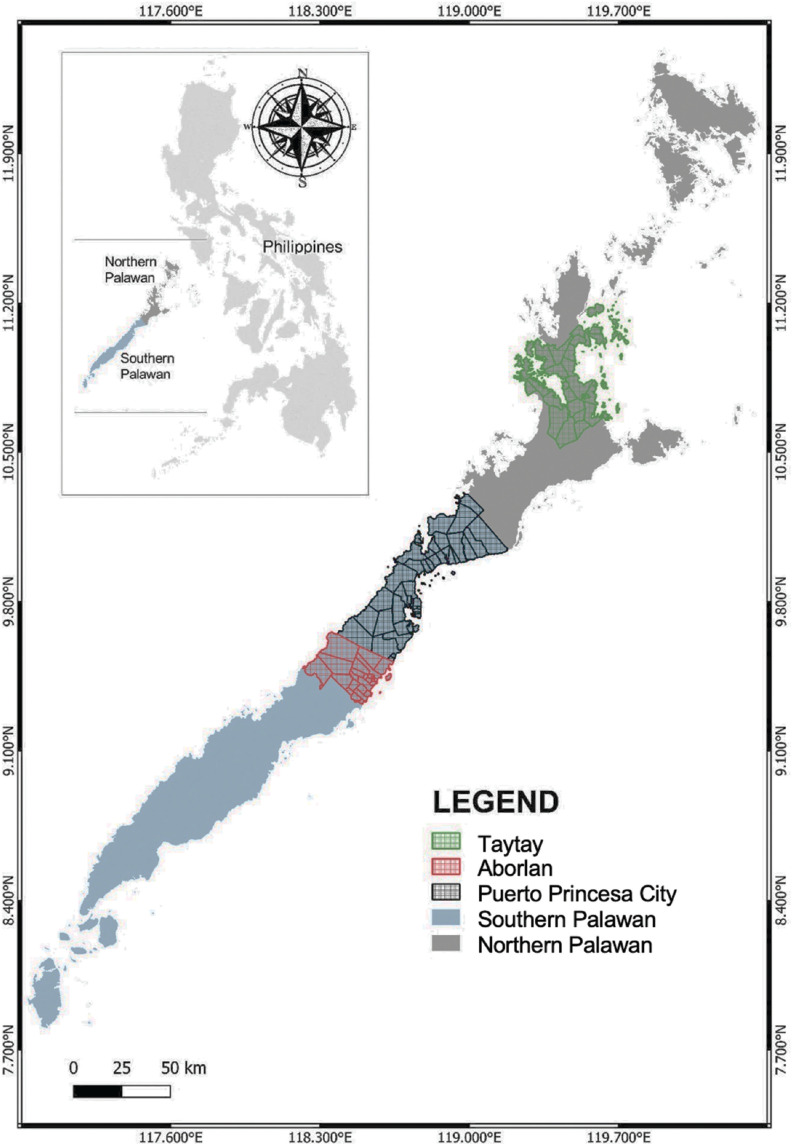
A map of Palawan, Philippines highlighting the southern and northern divisions and three of the GCRF BC’s case study areas – Puerto Princesa City and the municipalities of Taytay and Aborlan.

Palawan is a long and narrow island province in the Philippines (09°30′N and 118°30′′E) with an approximate total area of 1,489,626 ha and is located at the western portion of the archipelago (Fig. 1) [17,45]. Its almost 2000 km coastline is one of the longest shorelines in the country and accounts for about 1780 islands. The South China Sea borders the western coast while the Sulu Sea and the Malaysian Sabah Island border the eastern and southern sides of Palawan [46]. The island comprises 23 municipalities, one urbanised city (Puerto Princesa) and 433 small villages called ‘barangay’ [47].

Palawan is known as the Philippines’ ‘last ecological frontier’ due to its rich culture and biodiversity [48]. As per Presidential Proclamation No. 2152 of 1981, all mangrove forest areas in the province are protected as Palawan has been declared a Mangrove Swamp Forest Reserve [17,45]. In 1991, Palawan was designated as a biosphere reserve under the Man and the Biosphere Programme (MAB) of the United Nations Educational, Scientific, and Cultural Organization (UNESCO). The following year, the 1992 SEP Law assisted the MAB’s declaration in the sustainability of Palawan’s biological and cultural diversity. In succeeding years of recognising the biodiversity richness of the province, two out of nine UNESCO World Heritage Sites in the Philippines are to be found in Palawan: the Puerto Princesa Subterranean River National Park (inscribed in 1999) and the Tubbataha Reefs Natural Park (inscribed in 1993, 2009) [48].

Mangroves form one of the components of the coastal and marine ecosystems in the Philippines [49]. They are susceptible to various effects of climate change such as sea-level rise [50]. Therefore, adoption of various climate change adaptation interventions such as the National Framework Strategy on Climate Change [51] and the development of the Philippine exposure map on climate change [52] have been of great importance for the identification of vulnerable areas of Palawan that are the most susceptible to climate change.

**Figure 2:**
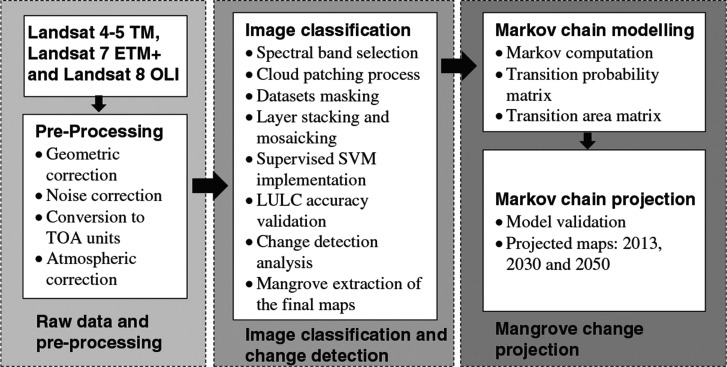
Diagram of multi-temporal mangrove change detection in Palawan using the Landsat imageries, supervised support vector machine classification and the Markov chain model.

The entire methodological process of mangrove classification and predictive modelling underwent three major processes: (1) raw data and pre-processing; (2) image classification and change detection; and (3) mangrove change projection (Fig. 2).

### Pre-processing the Landsat sensor data

The multi-temporal resolution and multi-spectral Landsat 4–5 Thematic Mapper (TM), Landsat 7 Enhanced Thematic Mapper Plus (ETM+) and Landsat 8 Operational Land Imager (OLI) images in multiple years between 1988 and 2020 were used for this study (Table A1 in Appendix A). A total of 20 scenes for TM (for years 1988, 1993 and 1998), 18 scenes for ETM+ (for years 2003, 2008 and 2013) and 11 scenes for OLI (for years 2018 and 2020) were sourced using the Semi-automatic Classification Plugin (SCP) version 7.9.0 Matera in Quantum Geographical Information System (QGIS) version 3.22.1 Białowiez˙a.

To normalise various conditions across the multi-temporal and multi-spatial Landsat datasets, it is imperative that Landsat data undergoes pre-processing routines to enhance the quality and remove various radiometric and geometric errors in each image [53–56]. Thus, radiometric calibration and atmospheric correction were employed for this study.

The 2018 OLI Level-2 data were used as the reference images to apply geometric correction to the satellite images in each epoch. The parameters of this transformation function were derived from a spread of 200 ground control points (GCPs), which were uniformly chosen from distinct topographic features throughout the target image. To match with the original pixel size of the Landsat data, all images were resampled to a ground resolution of 30 × 30 m and reprojected to WGS 84 UTM datum. The root mean square error (RMSE) of 0.25 pixel was calculated and was deemed enough to facilitate accurate LULC change detection analysis [57]. Throughout these processes, the nearest neighbour resampling algorithm was employed to maintain geometric integrity across all the images.

Radiometric correction followed the geometric correction [55]. Upon checking the image noise (e.g., dropouts and bit errors) for TM and ETM+ images using the Environmental Systems Research Institute’s ArcGIS version 10.7.1, a correction was not necessary. The next process of radiometric calibration involved the conversion of the signal of the quantified energy from multi-spectral brightness values or digital numbers (DNs) into top-of-atmosphere (TOA) reflectance units. In particular, this process involved two steps: (a) the conversion of DNs to spectral radiance (*L*_λ_) and (b) the transformation to TOA reflectance (*ρ*_λ_) as corrected for illumination variabilities (i.e., sun angle and Earth–sun distance) within and between scenes [55,56,58,59]. For the TM and ETM+ data, Eqs (1)–(5) were applied, respectively:

(1) $$L_{\lambda }=DN\times G+B$$

where *L*_λ_ corresponds to the radiance measured at the sensor bandwidth for each band (Wm^−2^sr^−1^*μ*^−1^); *DN* is the digital number value; *G* and *B* are the (gain) slope and (bias) intercept of response functions, calculated as follows:

(2) $$B=L_{\min}-(L_{\max}-L_{\min}/Q_{\max}-Q_{\min})\times Q_{\min}$$

(3) $$G=(L_{\max}-L_{\min}/Q_{\max}-Q_{\min})$$

where *L*_min_ and *L*_max_ are the lowest and highest radiance measured by a detector in mWcm^−^^2^sr^−^^1^, as reported by TM and ETM+ metadata files; *Q*_min_ and *Q*_max_ correspond to the minimum and maximum values of *DN* for TM and ETM+ sensors, ranging from 1 to 255. The TOA reflectance (*ρ*_λ_) calculation for each band applied on a pixel-by-pixel basis for each scene in each epoch and the output reflectance values were scaled to an 8-bit data range, this can be calculated as:

(4) $$\rho_{\lambda }=\frac{\left(\pi \times L_{\lambda }\times d^{2}\right)}{E_{0\lambda }\times \cos\theta_{s}}$$

where *d* is the Earth–sun distance correction; *L*_λ_ is the radiance as a function of bandwidth; *E*_0λ_ is the mean solar exoatmospheric irradiances and *θ*_s_ is the solar zenith angle. The application of absolute atmospheric correction and relative correction followed the corrections of sensor gains and offsets spectral band solar irradiance and solar zenith angle, and the topographic normalisation implementation. The removal of additive path radiance (*L*_p_) was calculated using Eq. (5) based on the dark-object subtraction (DOS) 1% technique [60–62]. The DOS assumes that the lowest reflectance value for dark objects across the image is 1% and any values greater than zero can be attributed to the additive effects of haze [41,55,63]. The relatively constant errors removal was implemented using the formula:

(5) $$L_{p}=L_{\min}+\left[\frac{\left(L_{\max}-L_{\min}\right)}{255}\right]\times DN_{\min}-0.01\times [(E_{0\lambda }\times \text{cos}\theta_{s}\times T_{z})+E_{\text{down}}]\times \frac{T_{v}}{\pi }$$

where *L*_p_ is the path radiance; *DN*_min_ has adopted the histogram technique [60] allowing the haze DN value to be automatically calculated from the DN frequency histogram of the image; and *T*_v_ and *T*_z_ are assumed to be equal in state thereby downward diffusion of radiation at the surface (*E*_down_ = 0) is absent [60].

### Spectral bands selection

In LULC classification, different land cover classes may respond to different ranges of wavelengths, and not all spectral bands are useful for the analysis. Consequently, it is imperative to appropriately identify the useful ranges of wavelength as the procedure increases class discrimination [64]. Chen et al. made an assumption that the low reflectance of mangroves in the short wavelength infrared (SWIR) region of the electromagnetic spectrum was due to the weak-scattering signal of the intercellular structure of the leaves [65]. Unsurprisingly, the low reflectance of the mixed mangrove assemblage with the surrounding mud and water could further reduce the reflected radiance of mangroves in general. Therefore, they used the Jeffries–Matusita distance technique to calculate the spectral separability among the LULC classes. This technique was adopted for this research and was conducted using the *spatialEco* package version 1.3–7 in R programming software [66–68].

The Jeffries–Matusita criterion measures the distance between the means of each class feature and the distribution of values around the means, giving a measure of spectral separability between the features of the class, and was thus able to determine the quality of the target class samples [68,69]. Values range from 0 to 2, where 2 indicates high separability while the lower values indicate a possible misclassification of the classes [70]. In the latter case, distances registered below the threshold of 1 were removed from the prioritised band image. Additionally, we have considered the Jeffries–Matusita values between 1.7 and 1.9, as good class separability [63]. In this study we combined the equivalent bands of each sensor to give an overall distance for the colour band. The generated results for the Jeffries–Matusita distance calculation indicate that the highest levels of separability between the mangrove vegetation and non-mangrove vegetation classes were observed for bands 5–4–3 for TM and ETM+ and 6–5–4 for OLI (Table 1). Thus, the band combination of SWIR1–NIR–Red was selected as the most appropriate band for the entire image classification.

**Table 1.** Spectral separability results using the Jeffries–Matusita distance technique to isolate the differences between the mangrove vegetation and non-mangrove areas for each band of TM, ETM+ and OLI sensors

TM bands	ETM+ bands	OLI bands	Band name	Jeffries–Matusita
1	1	2	Blue	0.51
2	2	3	Green	0.75
3	3	4	Red	1.63
4	4	5	NIR	1.86
5	5	6	SWIR 1	1.91
6	6	10	Thermal	0.72
7	7	7	SWIR 2	1.25

### Cloud patching process, stacking, mosaicking and masking

Clouds and cloud shadows have a significant effect on the satellite sensors’ spectral bands reflectance values [71] and degrade the quality of the sensors’ data [72]. Therefore, the Landsat database was searched for the clearest satellite images of the study area with the lowest cloud cover. However, for images where clouds are present, more than one scene from the same epoch was acquired to facilitate the cloud patching process using the Fmask algorithm [71,73]. The selection of different eras was based on the availability of quality data. Thus, the year 2021 was excluded from the potential list of options because most of the data available were poor in quality. All the selected bands were stacked together and created a seamless mosaic of the study area. The ocean areas were masked out using the Normalised Difference Vegetation Index with a threshold of cut-off of 0.5 [65].

### Image classification and change detection analysis

To delineate the mangroves of Palawan, this study used the support vector machine (SVM) classifier algorithm. This linear supervised non-parametric statistical learning theory has been proven effective in LULC research [74–76]. The SVM-based classifier requires a training sample and one of the advantages of this technique is that it can generalise well from a limited amount of training data compared to alternative methods [74]. This algorithm uses successive executions of a process until it generates the probabilistic estimates for known and unknown classes. In this entire procedure, the Bayesian minimum-error decision rule is adopted [77].

The overall accuracy results of SVM depend on the kernel used as well as the chosen kernel’s parameters and methods [78]. We chose the parameters gamma (G) in the radial basis function (RBF) kernel and the C hypermeter in SVM to control the error, using the cross-validation (CV) optimisation technique [79]. We set the default threshold values of 0.091 for G and 100 for penalty parameter C to gain a lower bias and penalise incorrect classification heavily [75]. The RBF kernel formula is shown below:

(6) $$K(x,x')=\exp(-g||x-x'|{|}^{2}),g>0$$

where ||*x* − *x*′||^2^ is the squared Euclidean distance between two data points, *x* and *x*′; *g* is the user-defined gamma. Across the series of Landsat data, we created two spectral classes including (a) mangrove vegetation, that is, intertidal halophytic forests both natural and rehabilitated, and (b) non-mangrove areas, for example, rivers, estuaries, lakes, sea, tidal mudflats, agricultural areas, grassland, high- and lowland forests, bushes, residential and industrial areas in rural and urban regions, aquaculture ponds, salt pans, etc. A random sampling technique was used to select a minimum of 400 pixels for each spectral class. For all the classified Landsat images, the total mangrove areas were quantified.

Assessing the accuracy of multi-decadal mangrove change is challenging due to the limited availability of in-situ reference datasets in the time period of interest [80]. In this work, the accuracy of mangrove classification was assessed using government data derived from the 2010 historical record of the National Mapping and Resource Information Authority (NAMRIA). The training mangrove forest polygons were validated through the established testing samples and the accuracy was assessed using the producer’s accuracy, the user’s accuracy, the overall accuracy and the kappa coefficient values [81]. This study produced >86% overall accuracy results by which the definite mapping identification of different land use/land cover categories generated valid results [82]. Furthermore, the kappa analysis for this study generated results >70%.

Upon completing the rigorous pre-processing, image classification and validation procedures, we conducted the change detection for Palawan and the three case study areas of GCRF BC, using the SCP version 7.9.0 Matera in Quantum in QGIS version 3.22.1 Białowiez˙a, to determine the magnitude of changes in mangrove vegetation and non-mangrove classes, and the trends of these changes across three time periods (1988–1998, 1998–2008 and 2008–2020).

### Mangrove change projection

A Markov chain is a stochastic process that describes the likelihood of changing one state to another [83] through the implementation of neighbourhood rules [84]. The Markovian process has been implemented in many LULC studies due to its efficiency in future land use prediction [40–42,85]. In mangrove forest spatial classifications, the integration of the Markov chain model [65] and its cross-functional application with cellular automata [85,86] is growing considerably.

In statistical terms, the Markov chain modelling can effectively make a prediction of the changes in LULC based on the calculation of the transition probabilities of one system at time *t*_2_ with the state of the system at time *t*_1_ according to the specific year [41,87]. The transition probability matrix [88] is one of the descriptive tools generated in the process where the mangrove areas transitional matrix is derived from different mangrove classes [86]. The Markov processes used in this study are expressed in Eqs (7–9):

(7) $$v_{t2}=Mv_{t1}$$

where the input LULC proportion column vector corresponds to *v*_*t*1_ and the output vector to *v*_*t*2_; *M* is an *m* × *m* transition matrix for the time interval Δ*t* = *t*_2_ − *t*_1_. The development of the probability transition matrix (*p*_ij_) can be calculated as follows:

(8) $$n_{i}=\sum_{j=1}^{q}n_{ij}$$

(9) $$p_{ij}=n_{ij}/n_{i}$$

where *n*_ij_ is the number of pixels of class *i* from the first date (current state) that were changed to class *j* in the second date (next period); cell *n*_i_ is in the change detection matrix by row marginal frequency; *q* is the total number of classified classes; and *p*_ij_ is the land-cover probabilistic transition matrix. We have conducted three projections using the Markov chain model. The first one was the mangrove projection for 2013 using the 1988–1993 datasets. In the second and third projection scenarios, we chose the years 2013–2020 datasets to predict the spatial changes of mangroves for the years 2030 and 2050. Using the IDRISI Environment version 17.00, the Markov chain transition probability matrix was generated.

## Model validation of the Markovian process

We validated the model by comparing the simulated mangrove and non-mangrove areas in 2013 with the observed data in the 2013 ETM+ map. The output was tested with observed values using the Pearson’s chi-squared (*χ*^2^) test to examine the appropriateness of the model:

(10) $$\chi^{2}=\sum \frac{{\left(O-E\right)}^{2}}{E}$$

where *O* represents the simulated value (1988–1993) and *E* is the actual value of the transition matrix (2013–2020). The calculated *χ*^2^ is compared with the *χ*^2^ from the table at alpha-level of 0.05 with (2 – 1)^2^ degrees of freedom. The land-use change analysis is compatible with the hypothesis of data independence if the computed *χ*^2^ is smaller than the tabled-value *χ*^2^.

## Results

### Spatiotemporal distribution of mangroves and comparison with the previous records

**Figure 3:**
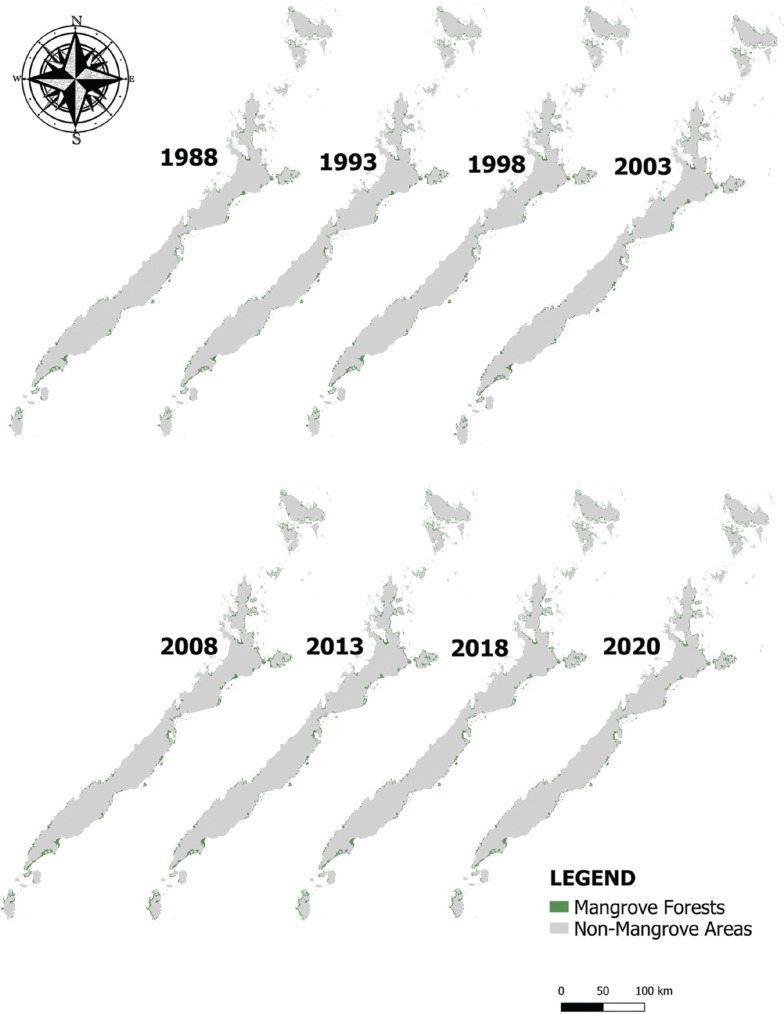
Spatiotemporal distribution of mangroves in Palawan in a span of 32 years from 1988 to 2020.

Our mapping classification resulted in two major classes, the mangrove forests and non-mangrove areas. We have presented in Fig. 3 the spatiotemporal distribution of mangroves in Palawan within the span of 32 years, particularly the time periods of 1988, 1993, 1998, 2003, 2008, 2013, 2018 and 2020. We observed that mangrove forests in Palawan were generally concentrated around the coastal boundaries, particularly in estuarine fringes, bays, riverbanks and the margins between land and sea. Based on this study and the previous records, the mangrove forest cover in Palawan was still relatively high compared with the other provinces in the Philippines (e.g., [18]).

The largest mangrove concentrations in Palawan were found in the eastern part of the island. These mangroves form dense and continuous stands in Puerto Princesa City (PPC), Bataraza, Balabac and Brooke’s Point in the south, and in the municipalities of Taytay, Coron, Busuanga, Culion, El Nido, Aracelli and Dumaran in the north. In PPC, the greatest concentration of mangroves is generally found in Puerto Princesa Bay, Honda Bay, Ulugan Bay and Turtle Bay.

The classified maps from 1988 to 2020 showed that the largest area of mangroves in Palawan was recorded in 2020 (60,033.8 ha) while the year 1998 (48,745.3 ha) had the least extent (Fig. 3). The lower total area calculated for 1998 is likely due to misclassification as a result of minor cloud patches, especially in the northern part of Palawan. Our estimate for this year, however, does not deviate too far from the estimates in 1993 (50,045.3 ha) and 2003 (52,961.5), respectively.

**Figure 4:**
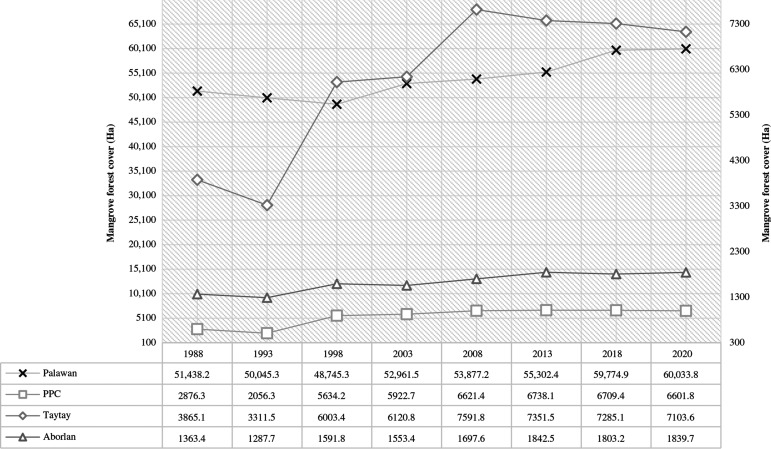
Composite representation of area statistics of mangroves in Palawan (left *y*-axis), PPC, Taytay and Aborlan (right *y*-axis).

In consideration of the funder of this study, we also separately quantified the mangrove extents in PPC, Aborlan and Taytay. Two of the GCRF BC’s smaller geographical case study areas (barangay) were located in Aborlan municipality while PPC and Taytay municipality both had four case study locations each. Among these three major boundaries, Taytay had the largest mangroves cover followed by PPC and Aborlan (Fig. 4). The mangrove areas in Taytay showed an increase since 1988 (3865.1 ha) and peaked in 2008 (7591.8 ha) before the trend showed a gradual decrease until the most recent estimate in 2020 (7103.6 ha). Similarly, the mangroves in PPC also exhibited a pattern of increase from 1988 (2876.3 ha) and reached the highest records in 2008 (6621.4 ha) and 2013 (6738.1 ha) before the total estimates dropped. Unlike the two previous locations, the mangrove forests in the municipality of Aborlan demonstrated an increasing trend from 1993 (1287.7 ha) to 2020 (1839.7 ha). However, the total mangrove area in Aborlan accounts for only about <25% and <30% of the overall mangrove forest covers in Taytay and PPC, respectively.

**Figure 5:**
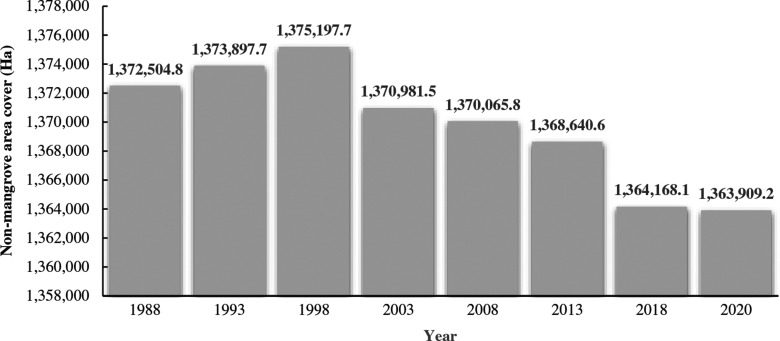
Estimated total cover of non-mangrove areas in Palawan from 1988 to 2020.

One of the most challenging aspects of classifying the non-mangrove areas in this study was the areal immensity of Palawan. The largest estimate for non-mangrove areas was recorded in 1998 at 1,375,197.7 ha (Fig. 5). Mainly, the non-mangrove areas identified were highland and lowland forests, agricultural areas and built-up areas (e.g., residential and industrial areas in rural and urban localities). A trend of decrease in non-mangrove areas was evident from 1998 to 2020 (1,363,909.2 ha). The smallest change, at approximately 250 ha, was recorded between 2018 (1,364,168.1 ha) and 2020.

**Figure 6:**
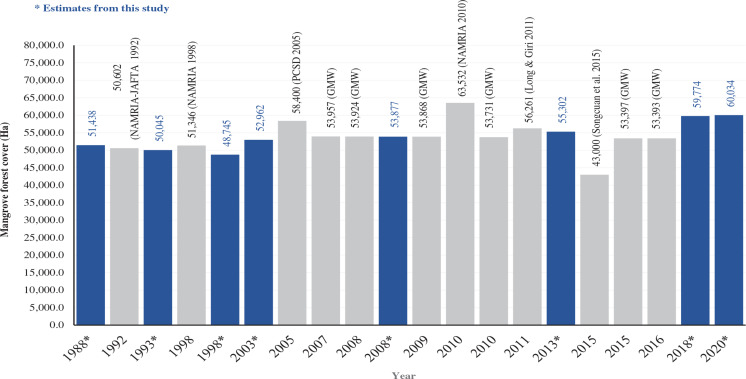
Representation of mangrove forest areas in Palawan based on the previous estimates (grey bars) and the results of this study (blue bars).

To visualise the mangrove forests extents in Palawan across the different time periods, which used different techniques and resources, the result of this study particularly for the years 2020, 2018 and 2013 were presented along with other previous estimates. As shown in Fig. 6, our estimates for the total areal extent of mangrove forests in Palawan are similar to other estimates from 1992 to 2015, except for the estimate of [89] at only 43,000 ha which was the lowest among all the gathered data. In the 1990s, the earliest records of mangrove estimates were obtained by the Japan Forest and Technology Association (JAFTA) [90] and NAMRIA. Our current estimate for 1993 (50,045.3 ha) was much lower compared with the previous records of DENR-JAFTA [90] and NAMRIA at 50,602 ha and 51,346 ha, respectively. However, our estimate for 1998 (48,745.3 ha) had about a 5% margin with the NAMRIA’s record (51,346 ha). In 2005, the PCSDS utilised the Satellite Pour I’Observation de la Terre (SPOT) satellite sensor’s images to delineate the extent of mangroves in Palawan and generated approximately 58,400 ha. Based on the mangrove data extraction made by Richter et al. [91] from the Global Mangrove Watch (GMW), in accordance with the same mangrove areal estimates that were originally created by Bunting et al. [92], the GMW figures from 2007 to 2010 had a very slight difference with the 2008 estimate (53,877 ha) for this study. Unsurprisingly, among all the references cited in this study, NAMRIA recorded the highest estimates at 63,532 ha in 2010 [15], which was higher than the GMW data in the same year (53,731 ha) and even higher than our most recent estimate for 2020. Our current study revealed a minor difference in the increase of mangrove forests, showing at least 59,774.2 ha in 2018 and 59,9925.8 ha in 2020, respectively (Fig. 6). Surprisingly, the mangrove forests assessment of Long and Giri [18] revealed a sudden decrease in mangrove areas in just a year span. Our estimates for 2013 at 55,302.4 ha had a minor margin of difference with the approximation obtained by Long and Giri [18].

The result of mangrove forest covers we obtained in 1993 (1287.7 ha) for Aborlan was comparably lower than the estimation made by [93] in 1992 (1494.8 ha). However, a small gap in the estimated values was determined between the work of Venturillo [93] in the same period and this study in 1998 (1591.8 ha; Table 2). Additionally, this study estimated the mangrove forests in Aborlan in 2008 at about 1676.6 ha which was higher than the GMW data (1341.3 ha). Although the interval of years was relatively small between 2010 and 2013, the assessment made by Jansen [94] in 2010 at 1202 ha was distinctly lower than the estimates from GMV [92] and our result for 2013 (1842.5 ha). Unsurprisingly, from the time periods 2013–2018, the GVM data for 2015 and 2016 [92] are similar, when in fact variations in areal changes were evident between 2013, 2014 and 2016. However, all the assessments reported for Aborlan revealed a similar pattern where mangrove forest cover increased from inclusive time periods 1992, 1993, 1998, 2010, 2013, 2014 and 2016.

**Table 2.** Comparison of mangrove forest areas in Taytay, Aborlan and PPC based on the previous estimates and the results of this study

Year (Reference)	Mangrove forest cover (Ha)		
	PPC	Taytay	Aborlan
1992 [93]	–	–	1494.8
1993	–	–	1287.7
1998	5634.2*	–	1591.8*
2003	5922.7*	–	–
2003 [27]	3201.8	–	–
2007 [91]	5839.8	6727.1	1340.7
2008 [91]	5835.7	6714.2	1341.3
2008	6621.4*	7591.8*	1697.6*
2009 [91]	5816.3	6713.2	1341.3
2010 [94]	4020.0	1578.0	1202.0
2010 [91]	5773.3	6715.5	1341.3
2013 [27]	4577.2	–	–
2013	6738.1*	7351.5*	1842.5*
2014 [93]	–	–	1866.8
2015 [91]	5754.8	6601.0	1337.2
2016 [94]	5668.0	3905.0	1655.0
2016 [91]	5754.8	6601.0	1337.2
2018	6709.4*	7285.1*	1740.3*
2020	6601.8*	7103.6*	1839.7*

The ‘*’ symbol denotes the estimates from this study. The GMW estimates were sourced from Richter et al. [91] and are based on the measurements by Bunting et al. [92].

In the municipality of Taytay, our estimated result obtained in 2008 has a close margin of difference from the GMW data. However, unsurprisingly our estimates for 2013 (7351.5 ha) and 2018 (7103.6 ha) differed significantly from the data gathered by Jansen [94] in 2010 (1578 ha) and 2016 (3905 ha; Table 2). A similar interpretation applies to the data by [94] in 2010 and from the GMW report in the same year where the former generated a very low estimate (1578 ha) against the latter figure of 6715.5 ha.

Pagkalinawan and Ramos [27] estimated the total mangrove forests extent in PPC at 3201.8 ha. It was less than our calculated results for 1998 (5634.2 ha) and 2003 (5922.7 ha), respectively (Table 2). On separate assessments, Jansen [94], Bunting et al. [92] and Pagkalinawan and Ramos [27] recorded 4020 ha, 5773.3 ha and 4577.2 ha of mangrove forests in 2010 and 2013. We obtained a relatively higher estimate in 2013 (6738.1 ha) compared with Pagkalinawan and Ramos [27] in the same year. We only observed an almost 100 ha difference between the estimates of Jansen [94] in 2016 and the quantified extent made by Bunting et al. [92] in the same year. However, between 2016 and 2020, an almost 1000 ha difference was observed between the previous and current estimates.

### Accuracy assessment

**Figure A1:**
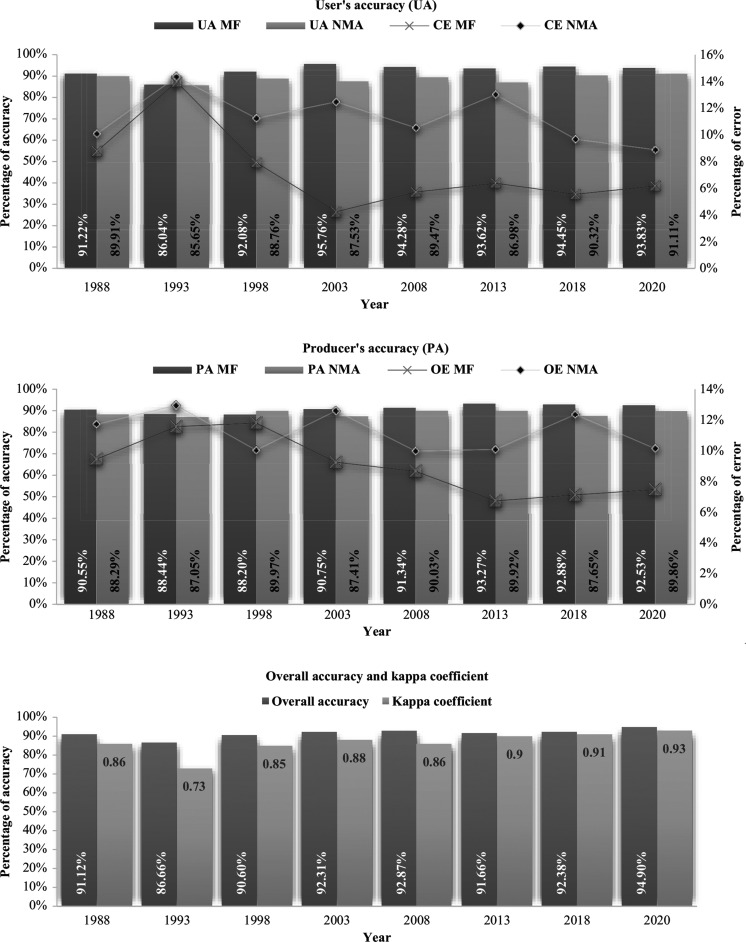
Classification error matrix of the Landsat TM, ETM+ and OLI data for multiple years, 1988, 1993, 1998, 2003, 2008, 2013, 2018 and 2020. The ground reference data used was the 2010 map derived from NAMRIA. The mangrove forests class is donated by ‘MF’ while the class of non-mangrove areas is denoted by ‘NMA’. Additionally, the measure of commission error (type 1 error) is denoted by ‘CE’ while the omission error (type 2 error) is denoted by ‘OE’, respectively.

Using the 2010 LU/LC NAMRIA map as our ground reference data, the mangrove classification accuracies for years 1988, 1993, 1998, 2003, 2008, 2013, 2018 and 2020 were generated. The comparative accuracy measurements yielded satisfactory agreements across all the years. The highest and lowest overall accuracies and kappa coefficient values for the mangrove forest class were produced in 2020 (92.90% and 0.91) and 1993 (86.66% and 0.73) classification maps, respectively (see Figure A1). The highest and lowest user’s accuracy in the classification of mangrove forest features were generated in the years 2003 (95.76%) and 1993 (86.04%). These suggest the commission errors of 4.24% and 13.96%, in which the pixels identified in the map as mangrove forest class actually represent an incorrect class based on a reference image. On the other hand, the generated producer’s accuracy quantifies the probability that a pixel was classified as something other than that class. The year 2013 yielded the highest producer’s accuracy (6.73% omission error) and the eras of 1998 and 1993 were at the lowest rank (11.80% and 11.56% omission errors). We presumed that the low overall accuracy and kappa coefficient values generated for 1993 were due to the poor satellite image quality. During this period, the cloud cover in two of the six scenes [refer to Table A2 in Appendix A: WRS Path 116/Row 052 (cloud cover = 3, cloud land cover = 13) and WRS Path 118/Row 054 (cloud cover = 8, cloud land cover = 20)] made marginal spectral confusion between different features. Generally, our classifications only produced <15% commission and omission errors for both mangrove forest and non-mangrove area classes (see Table A3).

### Mangroves change detection

We carried out change detection analysis for mangroves in Palawan by comparing multiple years in discrete intervals (e.g., 10-year gap, 7-year gap). The results of the change detection statistics within the four time periods (1988–1998, 1998–2008, 2008–2018, 2013–2020) showed that the mangrove extents in the Palawan dramatically increased for the last 32 years (Fig. 4, Table 3). The periods with the greatest change in mangrove forest extents in Palawan were recorded in 2008–2018 and 1998–2008, showing at least 10.95% (5897.7 ha) and 10.53% (5131.9 ha) increase since the time periods 1998–2018 (Table 3, Fig. 7a,b). However, we also noted the reduction in mangrove forest cover during the time period 1988–1998 at 5.24% (2692.9 ha) loss. Although this decrease might imply disturbance in the mangrove ecosystems in the study area, we did not exclude from our conclusion that this figure could be attributed to the spectral confusion of the different classes during the classification stage (see Table A3).

**Table 3.** Changes in mangrove forest distribution in Palawan during (a) 1988–1998, (b) 1998–2008, (c) 2008–2018 and (d) 2013–2020

Time period	Palawan		PPC		Taytay		Aborlan	
	Area (Ha)	%	Area (Ha)	%	Area (Ha)	%	Area (Ha)	%
1988–1998	2692.9^▽^	5.24^▽^	2757.9^▲^	95.88^▲^	2138.3^▲^	55.32^▲^	228.4^▲^	16.75^▲^
1998–2008	5131.9^▲^	10.53^▲^	987.2^▲^	17.52^▲^	1588.4^▲^	26.46^▲^	105.8^▲^	6.65^▲^
2008–2018	5897.7^▲^	10.95^▲^	88.0^▲^	1.33^▲^	306.7^▽^	4.04^▽^	105.6^▲^	6.22^▲^
2013–2020	4731.4^▲^	8.56^▲^	136.3^▽^	2.02^▽^	247.9^▽^	3.37^▽^	2.8^▽^	0.15^▽^

The percentage of reduction or increase in mangrove extents in each region was quantified based on the calculation used by [65]: (*S*_j_−*S*_i_)/*S*_i_ × *100*, where *S*_j_ and *S*_i_ represent the total areas in each categorical class in the *i*th and *j*th time periods. The symbol ‘^▲^’ denotes the percentage and areal increase in mangrove forests while the decrease is denoted by the symbol ‘^▽^’, respectively.

**Figure 7a:**
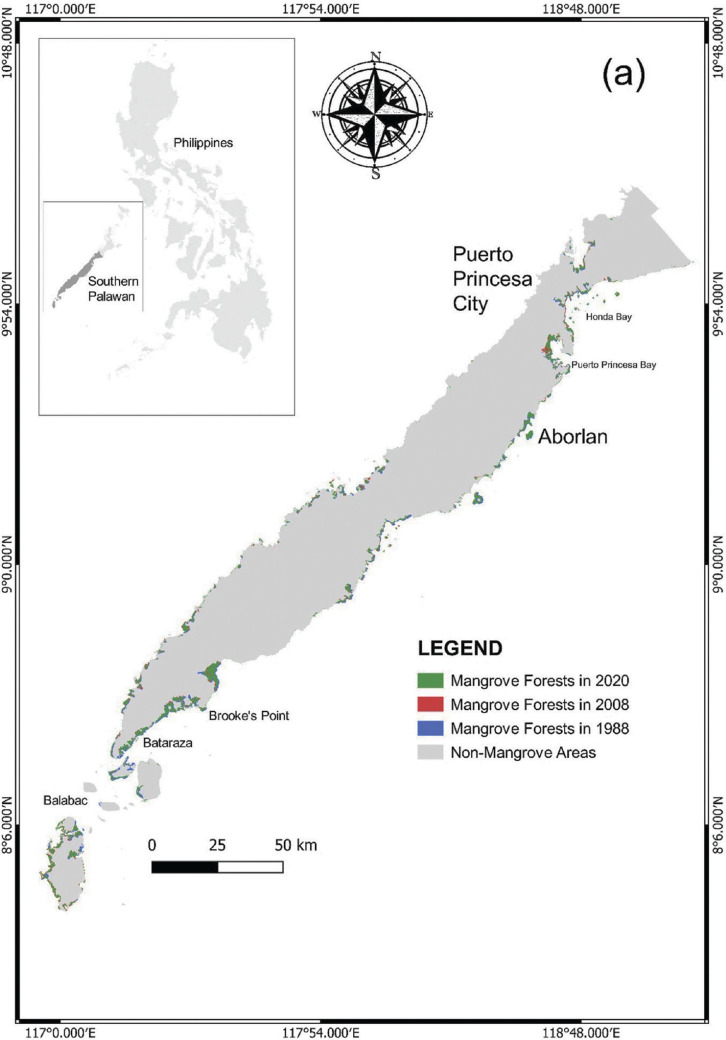
Changes in mangrove forests in Southern Palawan from 1988 to 2020.

**Figure 7b:**
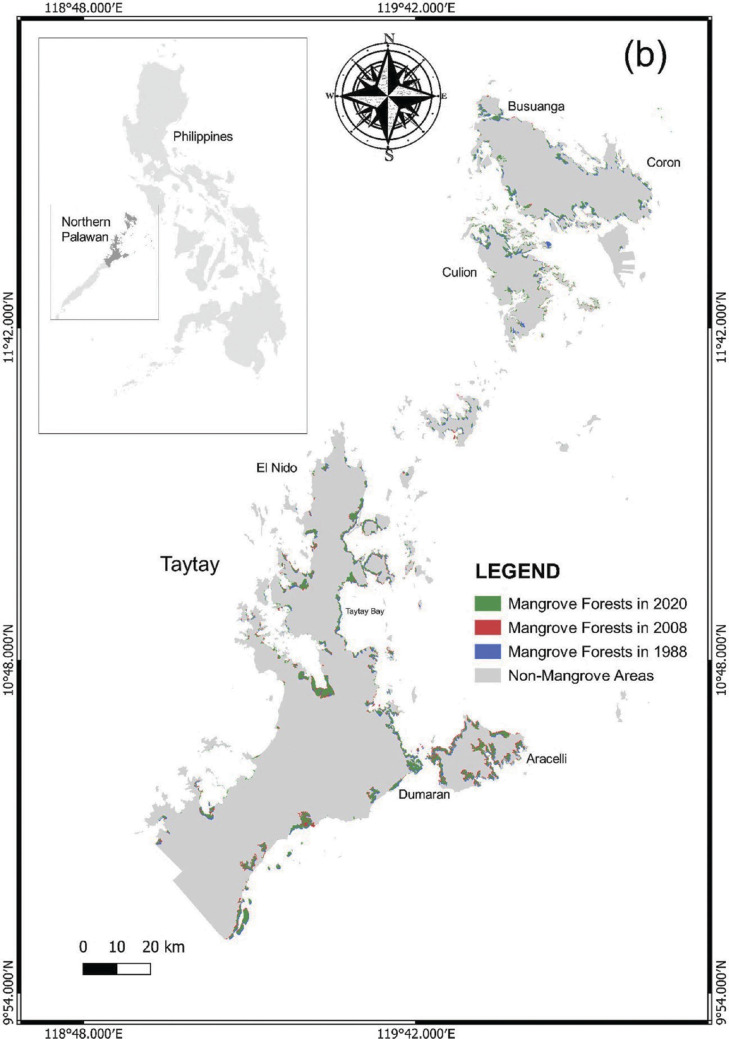
Changes in mangrove forests in Northern Palawan from 1988 to 2020.

Concurrently, the mangrove forest cover in PPC showed a sharp increase from 1988 to 1998 at about 2757.9 ha (95.88%). However, unlike the increasing trend in Palawan in 2013–2020, the percentage of change at 2.02% (136.3 ha) in the mangrove forest cover in PPC on the same time period showed a slight decrease. Most of the mangroves in PPC were found in the eastern seaboard of the study area, forming dense and narrow canopies along the riverbanks, estuarine regions and margins of the bays, particularly in Honda Bay, Puerto Bay and Turtle Bay. The only notable concentration of mangroves in the western seaboard of PPC was found in Ulugan Bay (Fig. 7c).

**Figure 7c:**
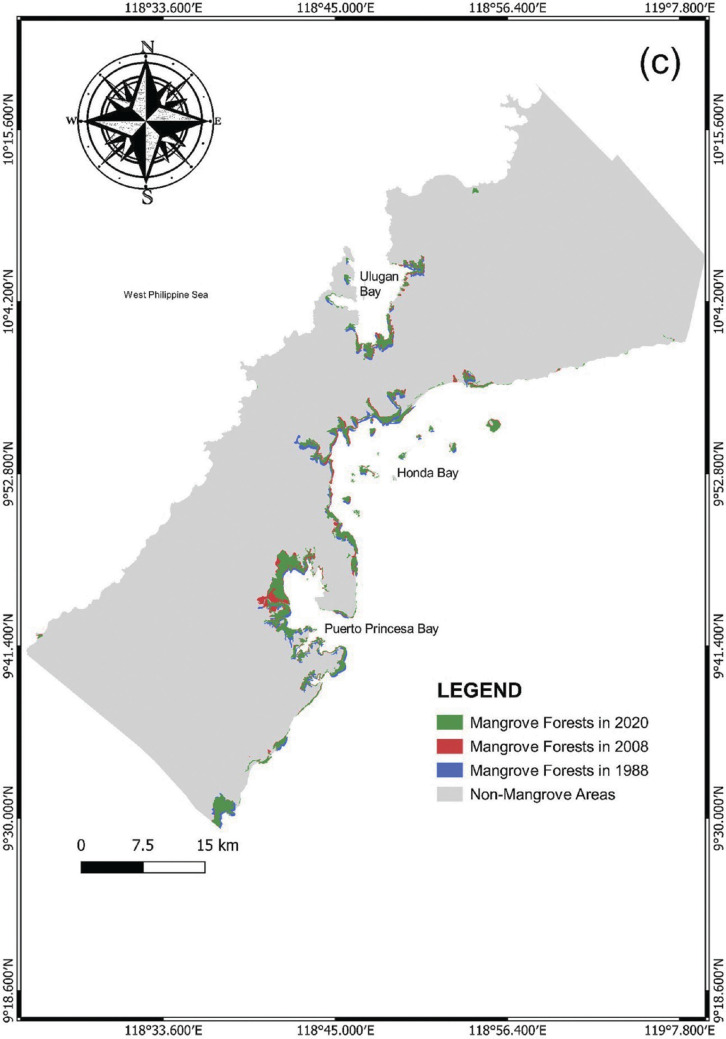
Changes in mangrove forests in Puerto Princesa City from 1988 to 2020.

Similarly, the municipality of Taytay also established an increase from the time periods 1988–1998 and 1998–2008 with the percentage of increase at about 55.32% (2138.3 ha) and 26.46% (91,588.4 ha), respectively (Table 3). Since 2008, the mangroves in this region suffered a consecutive loss, particularly with the reducing rates of 4.04% and 3.37% in 2008–2018 and 2013–2020, respectively. Despite this decrease, the mangrove extent in Taytay remained relatively higher than PPC and Aborlan (Fig. 3). These mangroves were mostly concentrated in Taytay Bay and along the Malampaya Sound area. The thick mangrove assemblages within the inner south-eastern portion of the Malampaya Sound were notable in the classified map. Furthermore, mangroves were seen forming boundaries along the coastlines of smaller and larger islands in Taytay Bay, especially in the north-eastern part of the bay (Fig. 7d).

**Figure 7d:**
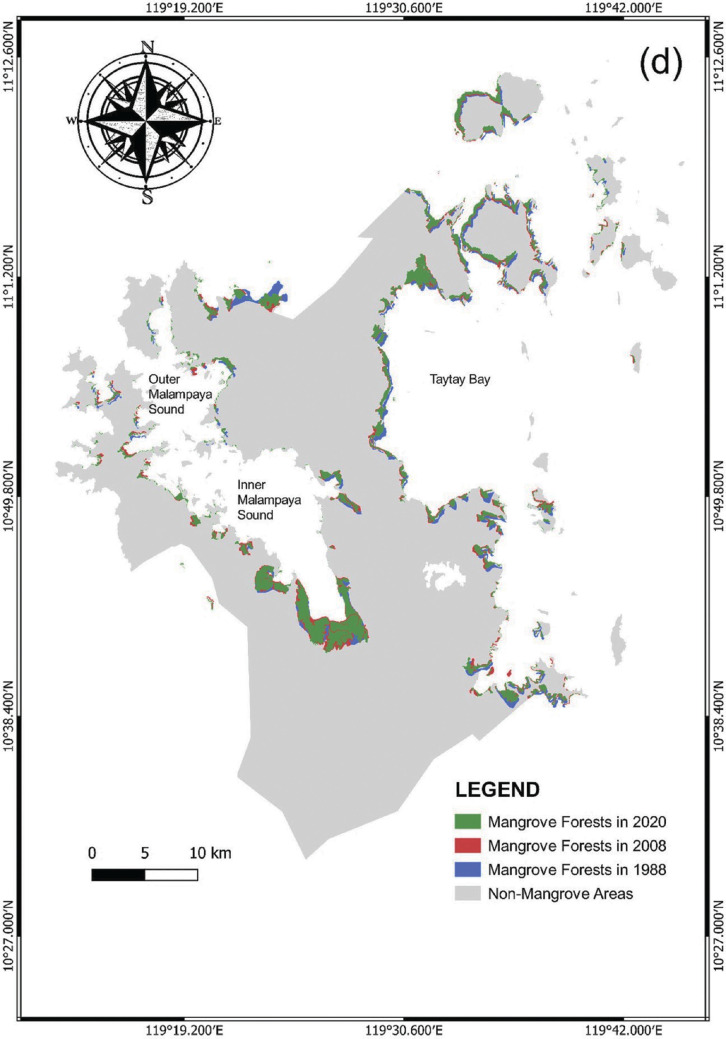
Changes in mangrove forests in Taytay from 1988 to 2020.

In comparison with the mangrove forests in Taytay and PPC, the municipality of Aborlan only suffered a small loss in mangrove assemblages during 2013–2020 (0.15%, 2.8 ha; Table 3). For the period of 20 years, the mangrove forest cover in Aborlan increased, although the extent of expansion was relatively lower than PPC and Taytay. Despite the similarities in the pattern of changes in Palawan, we did not exclude the possibility that the variations in tidal inundation and the time of the data acquisition may influence the estimations. Although we did not exclude the possibility that mangroves can also be found in the western seaboard of Aborlan, for this study we only recorded the mangroves in the eastern seaboard portion. Notably, the small islands of Puntog and Malunot generally had thick mangrove assemblages (Fig. 7e).

**Figure 7e:**
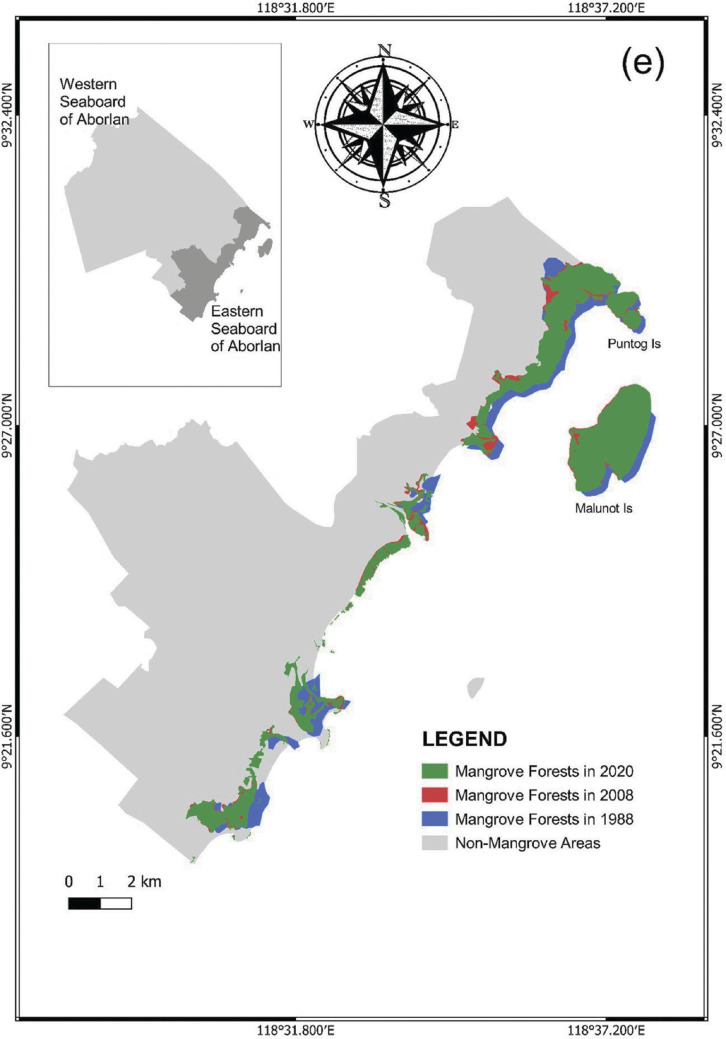
Changes in mangrove forests in Aborlan from 1988 to 2020.

There was a clear pattern of change in non-mangrove areas in Palawan from 1988 to 2020. An increasing trend was seen from 1988 to 1998 before a spike of decrease happened. The evidence of decreasing trend continued from 2003 to 2020 (Fig. 4). We assumed that these changes incorporate growth in closed-forest areas and the residential, industrial and agricultural developments in the region. Moreover, we also presumed that tourism growth and infrastructure expansion projects (e.g., construction of national roads or highways) play a critical role in the elaborated expansion of non-mangrove areas in Palawan.

### Mangrove forests projection and model’s accuracy

**Figure 8:**
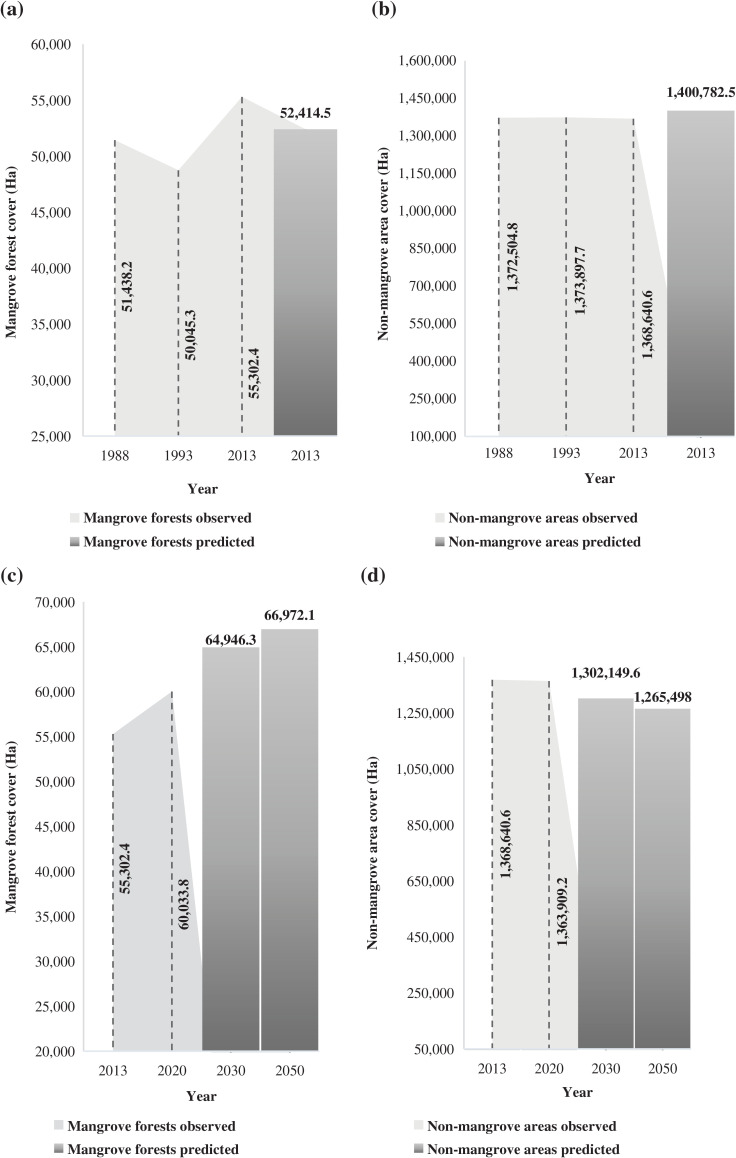
Projected probability of changes in mangrove forests and non-mangrove areas in Palawan. (a) Mangrove forests projection for 2013 using the time period 1988–1993. (b) Non-mangrove areas projection for 2013 using time period 1988–1993. (c) Mangrove forests projection for 2030 and 2050 using the time period 2013–2020. (d) Non-mangrove areas projection for 2030 & 2050 using the time period 2013–2020.

The Markov’s transition probability matrix was generated for the two time periods, 1988–1993 and 2013–2020 (see Appendix A). These numbers suggested the probabilities of change in mangrove forest and non-mangrove area classes in Palawan. The projected areal extent of mangroves for 2013 (52,414.5 ha) corresponds slightly with the observed 2013 extent at 51,438.2 ha (Fig. 8a), which indicated fewer variations between the two datasets. For this instance, we confirmed that the transition matrices between 1988 and 1993 could be effective for predicting the dynamics of change in the mangrove forests and non-mangrove areas in Palawan.

We found that the mangrove forests in the region will likely increase by 8.18% (64,946.3 ha) and 11.56% (66,972.1 ha) in the years 2030 and 2050 (Fig. 8c). Conversely, it was projected that the non-mangrove areas in Palawan were likely to reduce by 4.53% (1,302,149.6 ha) and 7.21% (1,265,498 ha) in 2030 and 2050, respectively (Fig. 8d). There was a slight increase in mangrove forests in Palawan for the simulated time period 2030 (64,946.3 ha) compared with 2013 (52,414.5 ha) and 2050 (66,972.1 ha; Fig. 8a,c).

The result of the accuracy assessment using the time period 1988–1993 and the projected 2013 output was evaluated using a *χ*^2^ test, indicating a value of 150.8 which was larger than 3.841 for the critical level of *P* = 0.05 with (2 – 1)^2^ degrees of freedom. This suggests that the hypothesis of statistical independence for the data was rejected. Therefore, predictive modelling using the Markov chain can be used for forecasting mangroves in Palawan.

## Discussion

The course of major development in Palawan was started in 1981 with the implementation of the Palawan Integrated Area Development Project [95]. Following the acquisition of Landsat data for 1988 in this study, this major project has been almost completed. Therefore, we deemed that this condition serves as a good baseline of information to envisage the changes in land use patterns in Palawan. But perhaps, the major framework for all development undertakings in Palawan was the passage of the Republic Act 7611 known as the SEP for Palawan Act in 1992. Within this law, the spatial basis for the implementation of its main goal is the Environmentally Critical Areas Network (ECAN) Zonation Project [96].

The strategic approach of ECAN is composed of three main components: terrestrial, coastal/marine zones and tribal ancestral lands. The multiple utilisations of every resource within these components are defined according to different zones, particularly within the multiple/manipulative zone and buffer zone. The buffer zone is further divided into three distinct zones where the level of restriction in resources extraction differs. The buffer zone comprises restricted use area (i.e., where limited non-consumptive activities may be allowed as long as they will not impair the ecological balance), controlled use area (i.e., activities such as mining, logging, tourism development, research and other minor resources extraction may be allowed to operate but must be strictly in compliance with the law) and traditional use area (i.e., located along the edges of intact terrestrial forests where traditional use has already been established). The intensive utilisation of land use in Palawan is clearly defined under the multiple/manipulative use zone areas [97,98]. Due to the ECAN zoning strategy, multiple land-use areas in Palawan have been assessed, marked and delineated based on their biophysical or natural and anthropogenic attributes to regulate activities, sustain the ecological integrity and properly manage the carrying capacity [45].

Gilman et al. [30] and Polidoro et al. [99] asserted that the economic growth and the augmentation of the human population are two major factors that influence the changes in the extent of mangrove forests and other land use areas. In PPC specifically, where the greatest housing development projects in Palawan are generally concentrated, the conception of the city’s housing project in 1992 had managed to transform different land use across its boundaries. For example, the multiple housing projects in Barangay Sicsican, Mangingisda, San Jose, San Manuel, Bahile, Tagburos, Sta. Cruz and Bahile, converted hundreds of hectares of collective land use areas into residential space. Although this number seems fairly alarming, the local government of PPC asserted that these initiatives could promote the smooth spatial expansion of the migration of mangroves in the future because most of the relocated local residents were previously living within the adjacent areas where mangroves are located [100].

Prior to the declaration of the protected area networks in Palawan, in 1981 and 1991, the mangrove areas in the province including the adjacent parcels of mangrove forests in the county were estimated at 74,267 ha [101]. Following the time after the integration of the SEP law in Palawan in 1992, the mangrove areas changed significantly [17] with at least 50,045 ha remaining areas in 1993 (Fig. 4). In contrast, a significant decrease of non-mangrove areas, which was notably recorded from this study from 1998 to 2018 (Fig. 5), coincides with the time periods where massive deforestation in the southern part of Palawan led to the reduction in the areal size of the forested areas during 2003–2010 [102]. Explicitly, we have found a significant increase in non-mangrove areas between 2013 and 2020, which was approximately 3 years after the implementation of the National Log Ban and the institutionalisation of an Anti-Illegal Logging Task Force in 2011. Interestingly, according to the report of DENR [103], among all the provinces in the Philippines, Palawan had the largest areal extent of forestland in 2020, totalling about 1,035,926 ha. We had identified that this study poses limitations against the generated results about the non-mangrove area class because we only referred to the generalisation of spectral separability. For this instance, we recommend that future similar studies should also focus on the spatial dynamics of multiple LU/LC areas.

Based on a joint venture initiative by NAMRIA and JAFTA in 1992, an aerial survey was conducted in Palawan. Among the notably remotely sensed information they obtained was evidence of small-scale logging activities, particularly in Taytay, and the slash and burn cultivation ‘Kaingin’ in the central boundary of PPC (e.g., Honday Bay, Ulugan Bay; Fig. 8b) and across the municipalities of San Vicente and Taytay [90]. PCSDS [96] further reported that a massive extraction of mangrove raw products for firewood consumption was rampant in Taytay. These anthropogenic stresses were assumed to cause changes in the land use/land cover areas in the northern part of the island during the pre- and post-establishment of a marine reserve within a small portion of the north-western tip of mainland Palawan (e.g., Bacuit Bay in El Nido municipality) in 1991.

However, following the expansion of the protected areas in northern Palawan (i.e., extension for 1991 – declared Bacuit Bay Marine Reserve) under the establishment of the El Nido-Taytay Managed Resources Protected Area in 1998 [104], the results obtained from this study (i.e., Fig. 7c), suggests a the reason for an increasing trend in mangrove forest cover in Taytay. Correspondingly, an approximately 8.7% increase in old-growth forest coverage in the protected area of Bacuit Bay has been reported a year after it become fully protected under the law in 1991 [105]. Moreover, PCSDS [96] reported that two endemic mangrove species in the Philippines namely, *Rhizophora stylosa* and *Compostenum philippinnensis*, were abundant in the northern part of Palawan including Taytay. For this reason, we assume that the abundance of their presence in this region contributes to the successful protection and recovery of mangrove forests.

Richter et al. [91] recently reported that communities interviewed generally perceived mangrove condition in Palawan had improved over the last 10 years. They reported that the perception of the local communities in Taytay, in reference with the mangrove forest ecosystem quality in their area, suggested no change in condition compared with the findings from this study that showed a decrease in extent over the past 10 years, although it is apparent that the extent has increased significantly over the interviewee’s lifetime [91]. Similarly, they reported that the communities in Aborlan and PPC perceived an improvement in mangroves over the last 10 years [91]. This study indicates that, while there was a gain in mangrove extent between 2008 and 2013, since 2013 there has been slight decline in mangrove cover or cover has remained stable in these areas (Fig. 6, Table 3).

The discrepancy in these results could be attributed to the reputation of Palawan for having still relatively high mangrove forest cover in comparison with the other provinces in the Philippines. The positive outlook of the local communities may be influenced by the environmental regulatory conceptions where they think that the province has strict regulated forest activities as the entire mangrove forests in the study area are located within the existing protected area networks (i.e., IUCN, SEP Law, ECAN Zoning Project). Also, because local communities were actively involved in yearly ‘mangrove tree planting’ activities across Palawan, for example, the local government of PPC has already planted around 800,000 mangroves since 2003 [106], they presume that this type of activity is a good indicator of a successful mangrove management. However, there were still no local studies that investigate whether the different mangrove rehabilitation programmes in Palawan are successful or not. It is also likely that, as this study used lower-to-moderate resolution satellite data, the ability to detect young mangroves that are small and sparce (i.e., saplings) is low so these areas may not be included in the extent figures. The perceptions of interviewees may also indicate improvements in mangrove condition and health, rather than simply on extent of mangrove coverage, which is information harder to attain by remote sensing.

On the other hand, we presumed that a large percentage of change in non-mangrove areas in Palawan could be attributed to the progressive changes of other ground features in the region (e.g., deforestation, forest regeneration, infrastructure, industrial and residential developments). For example, in PPC alone, a large portion of the non-mangrove area in the outskirt region of Barangay Sta. Lourdes, which was previously a part of higher elevated grassland/bushland region, has been converted into a sanitary landfill. Also, we have noted that the projected changes in the non-mangrove area class might be attributed to the mining activities in the southern Palawan, particularly in the municipalities of Bataraza, Brooke’s Point, Aborlan and Narra. Another contributing element, which we assumed could have a large contribution to the changes in non-mangrove areas in Palawan, was the inception of the Philippine government’s infrastructure-growth-targeting programme known as ‘Build! Build! Build’, which was started in the last quarter of 2016. Major highways, roads and bridges have been expanded or re-constructed across the country, including in Palawan, which led to the conversion of other land use areas. We expected that this type of development will continue to transform landscape patterns in Palawan until the end-term of the current government administration. Lastly, an increase in non-mangrove areas for the years 2030 and 2050 was also expected due to the influence of tourism demand in Palawan. As the global Covid-19 pandemic starts to shift to an endemic approach, the tourism industry in the province is now gradually gaining momentum. For example, this situation spurred global interest to visit/revisit the region’s historical and popular tourism sites, which had been restricted for almost 2 years due to the global outbreak of Covid-19.

The largest projection increment in mangrove aerial extents will be recorded in the next 30 years in 2050. We expected this evaluation following the assumption where the current ‘Build! Build! Build!’ programme of the Philippine government could catch up with rapid urbanisation and population growth, which could potentially facilitate the optimisation of mangrove forests protection in the province. This is because we assumed that relocating the local residents living within the coastal areas could lessen the threat to the mangrove ecosystem and foster community growth.

## Conclusions

Our study demonstrates the capability of the Markov chain model in predicting the future expanse of mangrove forests in Palawan using the multi-date Landsat satellite images from 1988 to 2020. This study found that in all study areas mangrove extent has increased from 1988 levels, although the trajectories since 2008 are more variable. Our analysis has shown the high likelihood of an increase in areal extent of mangroves in Palawan, from our most recent estimate in 2020 (60,033.8 ha) up to the years 2030 (64,946.3 ha) and 2050 (66,972.1 ha). However, these projections should be considered a baseline and must be interpreted with caution, as this work did not integrate environmental factors that may or had influenced the changes in mangrove forests. For the moment, it would still be sensible to accept that mangrove forests are under constant threat especially in the context of global climate change. The impact mechanism of sea-level rise on mangroves continues to increase as the greenhouse gas emissions persist. Furthermore, other threats such as coastal conversion, water pollution and raw products extraction are not slowing down and continue to potentially impact the mangrove ecosystems worldwide. Integrating mangrove forest projection at the regional scale is vitally important to determine specific resiliency response to climate change impacts.

The potential of the Markov chain model to project the potential changes of mangrove forests and other land use areas conveys its importance in the future, especially in the contexts of landscape management, ecological sustainability and policy intervention. However, as we did not create this type of model to directly assess our current policies, we recommend that future research should integrate the cellular automata–Markov model as it provides land cover data needed at different time steps (i.e., pre- and post-policy intervention) (e.g., [42]). This way, research bodies can evaluate the impacts of different policies (e.g., 1992 SEP Law, 1981 Mangrove Swamp Forest Reserve) in the future state of mangroves in Palawan. Furthermore, it would be good to conduct a similar study but it should also focus on the assessment of different LU/LC patterns to determine whether the demand of development that spurs the decrease or increase of certain features of non-mangrove areas is beneficial to the environment or not. This approach might alleviate uncertainties about the state of other multiple land-use areas in Palawan, other than mangrove forest, and the potential changes can be dissected and utilised for more effective management applications.

It would also be necessary to investigate the pressures of different socio-economic activities of village communities on the extent of mangrove forests within the different multiple zones (i.e., based on the ECAN Zoning Project) as changes in the distribution and intensity of these activities in response to social and economic drivers have the potential to contribute to changes in LU/LC areas. Given all the other driving factors that could influence the changes of mangrove forest cover in Palawan, we further encourage the implementation of spatio-statistical modelling techniques in the future, where the changes in land-use areas are to be fitted with environmental covariates. We think that this type of approach is timely, relevant, cost-effective and could enable the evaluation of different management interventions and policies not only in Palawan but also in the Philippines and neighbouring Southeast Asian countries.

## Data Availability

The datasets generated during and/or analysed during the current study are available from the corresponding author on reasonable request.

## Appendix

### Appendix A

#### Landsat sensors used

The multi-temporal resolution and multi-spectral Landsat 4–5 Thematic Mapper (TM), Landsat 7 Enhanced Thematic Mapper Plus (ETM+) and Landsat 8 Operational Land Imager (OLI) sensors were utilised for this study. The different ranges of frequencies along with the electromagnetic (EM) spectrum for TM, ETM+ and OLI are summarised in Table A1.

**Table A1.** Summary of band designations and spatial resolution for TM, ETM+ and OLI [111]

Sensor	Landsat 4–5 TM	Landsat 7 ETM+	Landsat 8 OLI	Spatial resolution
Coastal aerosol	–	–	B1 (0.43–0.45)	30 m
Blue	B1 (0.45–0.52)	B1 (0.45–0.52)	B2 (0.45–0.51)	30 m
Green	B2 (0.52–0.60)	B2 (0.52–0.60)	B3 (0.53–0.59)	30 m
Red	B3 (0.63–0.69)	B3 (0.63–0.69)	B4 (0.64–0.67)	30 m
NIR	B4 (0.76–0.90)	B4 (0.77–0.90)	B5 (0.85–0.88)	30 m
SWIR 1	B5 (1.55–1.75)	B5 (1.55–1.75)	B6 (1.57–1.65)	30 m
SWIR 2	B7 (2.08–2.35)	B7 (2.09–2.35)	B7 (2.11–2.29)	30 m
Thermal	B6 (10.40–12.50)	B6 (10.40–12.50)	B10 (10.60–11.19)	30 m
	–	–	B11 (11.50–12.51)	–
Pan-chromatic	–	B8 (0.52–0.90)	B8 (0.50–0.68)	15 m
Cirrus	–	–	B9 (1.36–1.38)	30 m

The empty cells correspond to the unavailability of the sensor for a particular feature. ‘B’ represents the band number and the corresponding wavelength range, enclosed in a parenthesis, and in a micrometre unit.

#### Sourced dataset

The TM, ETM+ and OLI datasets in multiple years 1988, 1993, 1998, 2003, 2008, 2013, 2018 and 2020 were sourced using the Semi-Automatic Classification Plugin (SCP) version 7.9.0 Matera in Quantum Geographical Information System (QGIS) version 3.22.1 Białowiez˙a (Table A2).

**Table A2.** Details of acquired Landsat satellite data selected for this study

Satellite sensor	Acquisition date (mm/dd/yy)	SRes (m)	WRS P/R	Satellite sensor	Acquisition date (mm/dd/yy)	SRes (m)	WRS P/R
TM	03/12/1988	30	115/053	ETM+	01/14/2003	30, 15	118/054
TM	01/31/1988	30	116/052	ETM+	01/23/2008	30, 15	115/053
TM	04/20/1988	30	116/053	ETM+	04/19/2008	30, 15	116/052
TM	06/30/1988	30	117/053	ETM+	10/12/2008	30, 15	116/053
TM	09/18/1988	30	117/054	ETM+	04/10/2008	30, 15	117/053
TM	01/29/1988	30	118/054	ETM+	10/03/2008	30, 15	117/054
TM	11/05/1993	30	115/053	ETM+	04/01/2008	30, 15	118/054
TM	12/14/1993	30	116/052	ETM+	10/19/2013	30, 15	115/053
TM	05/20/1993	30	116/053	ETM+	02/28/2013	30, 15	116/052
TM	10/27/1993	30	116/053	ETM+	05/19/2013	30, 15	116/053
TM	07/14/1993	30	117/053	ETM+	03/07/2013	30, 15	117/053
TM	06/12/1993	30	117/054	ETM+	06/27/2013	30, 15	117/054
TM	03/15/1993	30	118/054	ETM+	05/01/2013	30, 15	118/054
TM	11/10/1993	30	118/054	OLI	12/12/2013	30, 15	115/053
TM	01/03/1998	30	115/053	OLI	08/29/2018	30, 15	116/052
TM	03/31/1998	30	116/052	OLI	02/18/2018	30, 15	116/053
TM	03/31/1998	30	116/053	OLI	04/30/2018	30, 15	117/053
TM	01/17/1998	30	117/053	OLI	12/10/2018	30, 15	117/054
TM	01/17/1998	30	117/054	OLI	04/05/2018	30, 15	118/054
TM	02/09/1998	30	118/054	OLI	04/05/2020	30, 15	115/053
ETM+	04/15/2003	30, 15	115/053	OLI	09/19/2020	30, 15	116/052
ETM+	02/17/2003	30, 15	116/052	OLI	09/19/2020	30, 15	116/053
ETM+	02/01/2003	30, 15	116/053	OLI	08/25/2020	30, 15	117/053
ETM+	03/12/2003	30, 15	117/053	OLI	08/25/2020	30, 15	117/054
ETM+	04/13/2003	30, 15	117/054	OLI	05/12/2020	30, 15	118/054

For satellite sensors, the multi-spectral Landsat 4–5 is denoted by ‘TM’, the Landsat 7 Enhanced Thematic Mapper Plus is denoted by ‘ETM+’ and ‘OLI’ stands for Landsat 8 Operational Land Imager. The spatial resolution for each satellite image is denoted by ‘SRes’. ‘WRS’ means worldwide reference system, indicated in path ‘P’ and row ‘R’.

#### Accuracy assessment

Using the 2010 LU/LC NAMRIA map as our ground reference data, the mangrove classification accuracies for years 1988, 1993, 1998, 2003, 2008, 2013, 2018 and 2020 were generated (Fig. A1). The training mangrove forest polygons were validated through the established testing samples and the accuracy was assessed using the producer’s accuracy, the user’s accuracy, the overall accuracy and the kappa coefficient values [107].

#### Mangrove forests projection and model’s accuracy

Based on the calculation of the transition probabilities of one system at time *t*_2_ with the state of the system at time *t*_1_ according to the specific year [108–110], the Markov’s transition probability matrix was generated for the two time periods, 1988–1993 and 2013–2020 (Table A3).

**Table A3.** Calculated transitional probabilities during 1988–2020

Time period	Probability matrix	Mangrove forests	Non-mangrove areas
1988–1993	Mangrove forests	0.531	0.469
	Non-mangrove areas	0.401	0.599
2013–2020	Mangrove forests	0.548	0.452
	Non-mangrove areas	0.633	0.367
